# PD-L1+ CTCs Are Associated with Adverse Pathological Features and Unfavorable Prognosis in Bladder Cancer

**DOI:** 10.3390/diagnostics16121776

**Published:** 2026-06-09

**Authors:** Tianshuo Feng, Linjing Jiang, Juntao Zhuang, Lingkai Cai, Xiao Yang, Hao Yu, Haiwei Yang, Qiang Lu

**Affiliations:** Department of Urology, The First Affiliated Hospital with Nanjing Medical University, No. 300, Guangzhou Road, Gulou District, Nanjing 210029, Chinayangxiao2915@163.com (X.Y.);

**Keywords:** circulating tumor cells, PD-L1, bladder cancer, prognosis

## Abstract

**Backgrounds**: As one of the important liquid biopsy methods in recent years, circulating tumor cells (CTCs) have been proven to be valuable in judging metastasis and prognosis in various tumors. With the advent of immunotherapy, the expression of programmed death-ligand 1 (PD-L1) on CTCs has also attracted researchers’ attention, but its value in bladder cancer has not been fully explored. **Methods**: This study enrolled patients diagnosed with urothelial carcinoma who were treated in the Department of Urology, The First Affiliated Hospital of Nanjing Medical University from 2020 to 2023. Peripheral blood samples of the patients were collected to detect PD-L1+ CTCs. Meanwhile, the patients’ basic clinical characteristics, pathological data, and follow-up information were collected. The Kaplan–Meier method was used to estimate the overall survival (OS) and progression-free survival (PFS) of the patients, and the log-rank test was employed to evaluate the statistical differences. COX regression analysis and Firth penalized Cox regression analysis were adopted to assess the risk factors. Finally, according to the clinical or pathological characteristics, the effects of PD-L1+/PD-L1− CTCs on different subgroups of the population were evaluated, respectively. **Results**: According to the inclusion and exclusion criteria, a total of 109 patients were finally enrolled in this study, including 95 males and 16 females, with a median age of 68 years. The expression of PD-L1 on CTC was as follows: 19 patients were PD-L1+ CTC and 90 patients were PD-L1− CTCs. The results showed that the pathological grade of patients with PD-L1+ CTCs was significantly higher than that of patients with PD-L1− CTCs (*p* = 0.003). With a median follow-up time of 48 months (IQR: 45.8–49.5), prognostic analysis indicated that PD-L1+ CTCs were a risk factor for OS in bladder cancer patients (HR = 4.696 (1.477–13.596), *p* = 0.011). Subgroup analysis revealed that in patients with non-muscle-invasive bladder cancer (NMIBC) and in the subgroup of patients with high pathological grade, those with PD-L1+ CTCs exhibited significantly poorer prognosis in terms of PFS and OS compared with patients with PD-L1− CTCs. **Conclusions**: The presence of PD-L1+ CTCs in patients with bladder carcinoma may be closely associated with high-grade disease and poor OS.

## 1. Introduction

As one of the most prevalent malignancies in males and a highly common tumor of the urinary system, bladder cancer ranks among the highest in both incidence and mortality within this category [1]. Its clinical manifestations often include painless gross haematuria. However, a considerable proportion of patients already present with muscle-invasive or metastatic disease at the time of initial diagnosis. Moreover, carcinoma in situ of the bladder is associated with relatively high rates of progression and recurrence [2]. Even a diagnosis via imaging or invasive cystoscopy followed by appropriate surgical interventions such as transurethral resection of bladder cancer or radical cystectomy combined with chemotherapy or immunotherapy [3] does not fully avoid the risk that non-muscle-invasive bladder cancer (NMIBC) will remain prone to recurrence, and muscle-invasive bladder cancer (MIBC) generally portends a poor prognosis [4,5]. Therefore, it is of great importance to develop a detection method capable of enabling early identification of bladder cancer, which can provide reliable guidance and value for subsequent clinical intervention and long-term treatment.

Currently, traditional detection methods for bladder tumors mainly rely on non-invasive examinations, including computed tomography (CT) and magnetic resonance imaging (MRI) for direct visual assessment of tumor size, number, morphology, and borders [6], or involve obtaining tissue samples through invasive cystoscopy followed by pathological confirmation [7]. The former approach suffers from insufficient accuracy and potential harm from contrast agents, while the latter carries risks of trauma and imposing a significant economic burden. Compared to traditional tissue biopsy, liquid biopsy, as a novel tumor detection method, has been applied in many tumor fields. It mainly detects tumor traces by utilizing accessible body fluids from patients (usually blood), including circulating tumor cell (CTCs), circulating tumor DNA (ctDNA), circulating miRNAs, tumor-derived extracellular vehicles (EVs) and specific protein signatures released by tumors into the circulation. Liquid biopsy can improve the detection and management of tumors from a systemic perspective and at a microscopic level. And due to its capability of timely and sensitive tumor detection under various clinical conditions and in complex tumor environments, it has been applied in the prospective assessment of early-stage and metastatic tumors [8,9].

Serving as the important biomarkers in liquid biopsy, CTCs are individual cells or cell clusters present in the bloodstream. Studies suggest that CTCs undergo significant alterations at the genomic, proteomic, and transcriptomic levels. This is one of the key factors enabling them to mediate tumor metastasis and colonization [10,11]. Other research indicates that CTCs serve as potent biomarkers for tumor recurrence or metastasis [12,13]. In bladder cancer, the number of CTC correlates with increased tumor staging and greater myometrial invasion [14]. Moreover, CTCs demonstrate considerable potential in predicting recurrence in high-risk NMIBC [15], assessing RC prognosis, and guiding adjuvant chemotherapy decisions for bladder cancer [16]. Our preliminary research also indicates that the quantities of solitary CTCs, small-cell CTCs, and tetraploid CTCs can predict sensitivity to neoadjuvant chemotherapy (NAC) in bladder cancer. Incipient patients exhibited a higher level of triploid and tetraploid CTCs than relapse patients [17]. However, with the advent of immunotherapy, researchers’ understanding of CTC subtypes with specific immunomodulatory functions remains inadequate. Therefore, investigating CTCs has significant implications for understanding the complex process of metastasis.

PD-L1, as the primary target for current immune checkpoint inhibitors, remains a focal point of research in the field of immunotherapy. As a camouflage signal from cancer cells, PD-L1 induces T cells to cease attack, creating an inhibitory immune microenvironment that ultimately enables immune escape [18]. Recent clinical studies suggest that PD-L1 expression in tissue appears unrelated to the complete pathological response rate following neoadjuvant chemotherapy and immunotherapy [19], which challenges its utility as a potential biomarker for immunotherapy. Kong D et al., through meta-analysis, found PD-L1 expression in peripheral blood CTC to be significantly associated with poor prognosis in tumor patients, with a pooled HR of 1.85 for OS and 1.50 for PFS [20]. In vulvar carcinoma, adjuvant chemotherapy, adjuvant chemoradiotherapy, and neoadjuvant chemoradiotherapy all lead to an increase in the proportion of PD-L1+ CTCs, and a certain number of PD-L1+ CTCs leads to poor PFS [21]. By conducting baseline assessments and continuous monitoring of PD2212L1 expression in CTCs, another research showed that in bladder cancer, over 90% of patients who are non-responders to Bacillus Calmette-Guérin (BCG) therapy, which is widely used for the treatment of non-muscle-invasive bladder cancer, exhibit PD-L1+ CTCs prior to treatment [22]. However, the value of PD2212L1+ CTCs in the clinical–pathological characteristics, prognosis, and immunotherapy response of bladder cancer remains to be fully explored.

In this study, we conducted CTC detection on bladder cancer patients at our center between 2020 and 2023, whilst simultaneously assessing PD-L1 expression within these CTCs. This investigation aimed to clarify the correlation between the expression status of PD-L1 in CTCs and pathological characteristics in bladder cancer patients, and to investigate the predictive value of this marker for clinical prognosis in the overall bladder cancer population and in patients with different bladder cancer subgroups.

## 2. Materials and Methods

### 2.1. Study Design

From September 2020 to May 2023, a total of 161 peripheral blood samples were prospectively collected from patients at The First Affiliated Hospital of Nanjing Medical University. Among these, 40 samples were excluded from further statistical analysis due to the following reasons: (1) unqualified sample quality that failed circulating tumor cell (CTC) detection (*n* = 8); (2) a previous medical history of other malignant tumors (*n* = 13); (3) repeated blood samples from the same patient (*n* = 6); (4) postoperative pathological diagnoses inconsistent with bladder urothelial carcinoma (*n* = 11); and (5) unavailable pathological results (*n* = 2). Ultimately, a total of 121 patients were included for clinicopathological characteristic analysis (Table S1). Of these 121 patients, a total of 109 presented positive CTC results (CTC ≥ 1). These 109 patients were further divided into two groups based on the positive (PD-L1 ≥ 1) or negative (PD-L1 = 0) expression of PD-L1 on the surface of CTCs, and the correlations between CTC PD-L1 status and a series of clinicopathological characteristics were subsequently analyzed. In the subsequent survival analysis, 22 patients were further excluded due to loss in follow-up, and considering that no endpoint events were observed in OS analysis and only one endpoint event occurred in PFS analysis in the CTC− subgroup (*n* = 10), CTC− patients were excluded, and only CTC+ patients (*n* = 89) were included in the present analyses. At admission, peripheral blood samples were collected for CTC detection. This study was approved by the Ethics Committee of the First Affiliated Hospital of Nanjing Medical University (2017-SRFA-016) and conducted in accordance with the Declaration of Helsinki. All patients provided written informed consent.

### 2.2. CTC Enrichment, Identification, and Classification

The detection of CTCs was consistent with previous methods [17]. In brief, we employed SE-iFISH technology to identify CTCs. SE-iFISH is a new approach to detecting CTCs that combines differential phase enrichment, tumor-labeled immunofluorescence staining, and i-FISH techniques. Peripheral blood samples were collected from all enrolled patients before surgery or one day prior to neoadjuvant chemotherapy after admission. A total of 6 mL of cubital vein blood was harvested between 6:00 and 6:30 in the early morning using an 8-gauge needle (SHANDONG WEIGAO BLOOD PURIFICATION PRODUCTS, Weihai, China) and ethylenediaminetetraacetic acid (EDTA)-containing anticoagulant blood collection tubes (Improve Medical Instruments Co., Ltd., Guangzhou, China). The initial 3 mL of blood was discarded to avoid contamination. All blood samples were stored at room temperature and delivered for testing within 24 h after collection. Blood samples were centrifuged at 200× *g* for 15 min (Eppendorf AG, Hamburg, Germany) at room temperature to separate plasma. The sedimented blood cells were mixed with 3.5 mL of hCTC buffer (Cytelligen, San Diego, CA, USA) and loaded onto a non-hematopoietic cell separation matrix (Cytelligen, San Diego, CA, USA) in a 50 mL tube (LabServ, DE, USA), followed by centrifugation at 450× *g* for 5 min. The solution containing WBCs and tumor cells above the RBCs was collected and subsequently incubated with 300 μL of immuno-magnetic beads conjugated to a cocktail of anti-leukocyte mAbs (Cytelligen, San Diego, CA, USA) at room temperature for 30 min. WBCs bound to immuno-beads were depleted using a magnetic separator (Cytelligen, San Diego, CA, USA). The sedimented cells were gently resuspended, mixed with cell fixative (Cytelligen, San Diego, CA, USA), smeared on formatted and coated CTC slides (Cytelligen, San Diego, CA, USA), and dried for subsequent iFISH processing. The dried monolayer cells on the coated slides were rinsed and incubated with PBS (Servicebio, Wuhan, China) at room temperature for 3 min, followed by hybridization with Vysis chromosome 8 centromere probe (CEP8) Spectrum Orange (Abbott Laboratories, Chicago, IL, USA) for 4 h. These were subsequently incubated with the indicated post-fluorescence labeled monoclonal antibodies at 1:200 dilution, including Alexa Fluor (AF)594-CD45 (ATCC, Clone 9.4, Manassas, VA, USA), AF488-PD-L1 (Dana-Farber Cancer Institute, Clone 29E.2A3, Boston, MA, USA), and Cy5-CD31 (Abcam, Clone EP3095, Burlingame, CA, USA), at room temperature for 20 min in dark. After washing, samples were mounted with mounting media containing DAPI (Vector Laboratories, Burlingame, CA, USA). The indicated hepatocellular carcinoma cells (HCC) HepG2 (ATCC, VA, USA) and non-small cell lung cancer cells A549 (ATCC, VA, USA) were co-examined by immunofluorescence (IF) staining as control cell lines. Coated slides containing aneuploid CTCs stained by iFISH were scanned by means of an automated Metafer-i•FISH^®^ CTC 3D scanning and image analyzing system (co-developed by Carl Zeiss, Oberkochen, MetaSystems, Altlussheim, and Cytelligen, San Diego, CA, USA). CTC slides were automatically loaded onto a Zeiss fluorescence microscope (AXIO Imager Z2), and afterwards subjected to automated X-Y scanning with cross Z-sectioning of all cells performed at 1 μm steps of depth. X-Y-Z 3D scanning was performed in each of the 5 fluorescence color channels.

**Figure 1:**
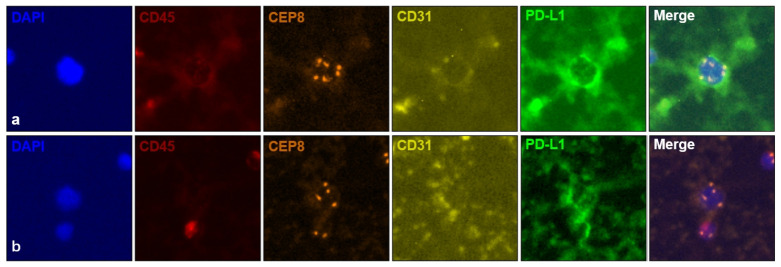
Identification of CTCs by SE-iFISH. Line (**a**): PD-L1 positive (PD-L1+) CTCs. Line (**b**): PD-L1 negative (PD-L1−) CTCs. Blue indicates DAPI, red indicates CD45, orange indicates CEP8, yellow indicates CD31, and green indicates PD-L1.

The identification criteria of CTCs were as follows: aneuploid chromosome 8 with nucleus DAPI+/CD45−/CD31−. We invited two independent pathology experts to ensure the accuracy of fluorescence image interpretation. We rigorously assessed and recorded the presence of CTCs and the status of PD-L1 expression on CTCs in each enrolled patient, forming the foundation for subsequent analyses (Figure 1).

### 2.3. Follow up

We used the outpatient service and phone calls for follow-up. Patients with RC were usually seen every 3 months following surgery during the first year, and every 6 months from the second to fifth years. Every 6 months, patients would take abdomen imaging, including but not limited to the CT or MRI of the abdomen or pelvis with intravenous contrast of urinary tract, together with a chest radiography after surgery. For TURBT, patients received an additional cystoscopy and urinary cytology every 3 months in the first year following surgery and every 6 months from the second to fifth years. In this process, we employed a variety of methods to gain related information, including medical history taking, serum, urine chemistry evaluation, and also physical examinations. The follow-up period commenced from the date of the patient’s initial surgery. PFS was defined as the time from the start of surgery to the occurrence of radiological progression or death from any cause, and OS was defined as the time period between operation and death from any cause. All patients were followed up on time.

### 2.4. Statistical Analysis

Continuous variables were presented as median with interquartile range (IQR), and categorical variables were reported as absolute numbers and percentages. For comparative analyses, categorical variables were examined using the Fisher’s exact test or the χ^2^ test, as appropriate. Continuous variables were compared with the Student’s *t*-test or the Mann–Whitney U test, depending on data distribution. The Kaplan–Meier method was used to estimate the OS and PFS of the patients. Univariate and multivariate Cox proportional hazards regression models were applied to calculate HRs along with 95% confidence intervals. These factors included age, gender, PD-L1 expression status in CTCs, presence or absence of myometrial invasion, lymph node metastasis, special histological subtypes, lymph vascular Invasion (LVI), pathological grade, tumor size, tumor number, neoadjuvant therapy, maintenance intravesical instillation, adjuvant systemic treatment, surgical modality, and pathological staging.

Furthermore, group differences were evaluated with the log-rank test to investigate the prognostic value of PD-L1 expression on CTCs in bladder cancer patients stratified by different pathological characteristics. Univariate Cox regression analyses were first performed for OS and PFS, and variables with a *p*-value < 0.05 were subsequently included in the multivariate analyses. Given only 22 PFS events and 17 OS events, a limited sample size and potential complete data separation were observed, which compromised the reliability of conventional maximum likelihood estimation. Therefore, Firth penalized Cox regression analysis was utilized in the multivariate analysis to obtain stable estimates by incorporating the Jeffreys prior penalty, which is well suited for small-sample and sparse-data scenarios.

All statistical analyses were performed with SPSS software (version 25.0) or the coxphf package in R 4.4.0. A two-sided *p* value < 0.05 was considered statistically significant.

### 2.5. Quantitative Distribution and Grouping Criteria of CTCs and CTC Surface PD-L1 Expression

Among the 121 enrolled patients included in the final analysis, CTC positivity was defined as a CTC count ≥ 1, and CTC negativity was defined as a CTC count of 0. A total of 109 (90.1%) patients presented CTC+, while 12 (9.9%) patients were CTC−. The maximum CTC count was 89, with a median count of 4. For the evaluation of CTC surface PD-L1 expression, PD-L1 positivity was defined as CTC+ cases with ≥ 1 PD-L1-expressing CTC, and all remaining CTC+ cases were classified as PD-L1−. After excluding CTC− individuals, 109 patients were included for subsequent analysis. Among them, 19 (17.4%) patients were identified as PD-L1+ CTC, and 90 (82.6%) were PD-L1− CTC. In patients with positive PD-L1 expression on CTCs, the maximum number of PD-L1+ CTCs was 16, with a median value of 1.

## 3. Results

### 3.1. Clinical and Pathological Characteristics of Patients

**Figure 2:**
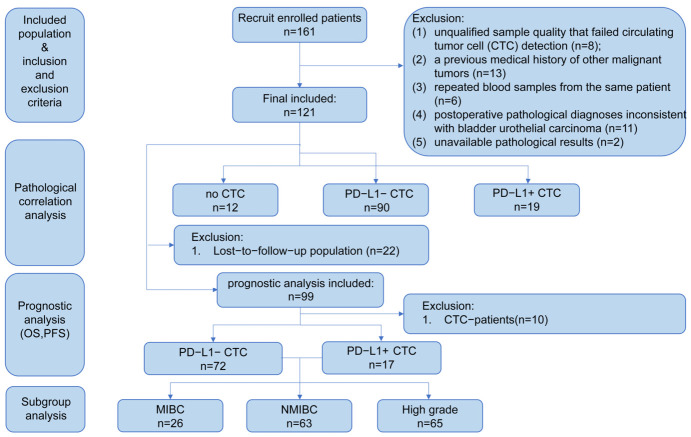
Flowchart of this study.

This study included a total of 161 patients. The patient enrollment and grouping flowchart of this study is shown in Figure 2. Following inclusion and exclusion criteria, a total of 121 patients were included in the analysis, comprising 103 males and 18 females, with a median age of 66 years. We stratified the cohort into two groups based on the presence or absence of circulating tumor cells (CTCs) and performed a correlation analysis of clinicopathological characteristics (Supplementary Table S1). The results indicated that no significant differences were observed in the correlations of these clinicopathological features between CTC− and CTC+ patients. In all, 109 patients were ultimately analyzed (Table 1). Among these, 93 were male and 16 were female, with a median age of 68 years. Patients with PD-L1+ CTCs numbered 19 (17.4%), and PD-L1− CTCs numbered 90 (82.6%). In addition, there were 28 (25.7%) patients with low-grade pathological grade and 81 (74.3%) with high-grade pathological grade. NMIBC patients numbered 73 (70.0%), and MIBC numbered 36 (33.0%). Nine (8.3%) patients had lymph node metastasis. Furthermore, there were 15 (13.8%) patients with specific subtypes of bladder cancer (not urothelial carcinoma), including squamous cell carcinoma, mucinous adenocarcinoma, and so forth. Moreover, the pathological findings of the bladder cancer indicated 10 (9.2%) patients had LVI. For the analysis of the correlation between pathological features and PD-L1 expression on CTCs, patients were divided into a primary tumor-maximum diameter ≥ 3 cm group (*n* = 29) and a primary tumor-maximum diameter < 3 cm group (*n* = 80) according to the results of preoperative MRI or CT. The grouping criterion for Tumor Multifocality was the presence of ≥2 independent and separate tumor lesions as determined by imaging and pathological results. Based on this, patients were categorized into a multifocal group (*n* = 42) and a unifocal group (*n* = 67). Pathological tumor-node-metastasis (TNM) staging was assessed by pathologists according to the Union for International Cancer Control/USAn Joint Committee on Cancer (UICC/AJCC) 8th edition pathological TNM staging criteria for bladder cancer. The overall clinical stage was categorized as Stage 0 to IV, and patients were grouped accordingly. In the PD-L1− CTCs group, there were 44 (95.7%) patients with Stage 0 disease, 18 (69.2%) with Stage I, 12 (75.0%) with Stage II, 8 (88.9%) with Stage III, and 8 (66.7%) with Stage IV. In the PD-L1+ CTC group, there were 2 (4.3%) patients with Stage 0 disease, 8 (30.8%) with Stage I, 4 (25.0%) with Stage II, 1 (11.1%) with Stage III, and 4 (33.3%) with Stage IV. We combined Stage 0 and Stage I into the low pathological stage group (a total of 62 patients with PD-L1− CTCs and 10 patients with PD-L1+ CTCs), and the remaining pathological stages were uniformly included in the high pathological stage group (a total of 28 patients with PD-L1− CTCs and nine patients with PD-L1+ CTCs).

In the survival analysis, considering the potential impact of treatment factors, we also included Neoadjuvant therapy, Maintenance intravesical instillation, and Adjuvant systemic treatment in the subsequent analyses. For the Neoadjuvant therapy (NAT) variable, the grouping criterion was whether patients received systemic pharmacological therapy before surgery, such as the gemcitabine plus cisplatin (GC) regimen. Based on this, patients were divided into a preoperative treatment group (*n* = 11) and a no preoperative treatment group (*n* = 78). The grouping criterion for Maintenance intravesical instillation (MII) was whether patients received more than one bladder instillation treatment after surgery (as patients with bladder cancer in our center routinely receive one postoperative instillation). Accordingly, patients were classified into a postoperative instillation group (*n* = 33) and a no postoperative instillation group (*n* = 56). Finally, for the Adjuvant systemic treatment (AST) variable, the grouping basis was whether patients received systemic pharmacological therapy after surgery, such as the gemcitabine plus cisplatin (GC) regimen. Based on this, patients were divided into a postoperative systemic treatment group (*n* = 18) and a no postoperative systemic treatment group (*n* = 71).

### 3.2. PD-L1+ CTCs Correlate with High Pathological Grade in Bladder Cancer

A total of 121 patients were divided into a CTC− group (*n* = 12) and a CTC+ (*n* = 109) based on the presence or absence of CTCs. Correlation analyses between these two groups and clinicopathological variables revealed no significant differences between CTC− and CTC+ patients for any of the included clinical variables (Table S1).

Among the 109 patients with CTCs, the cohort was divided into two groups based on PD-L1 expression status of CTCs. We divided the enrolled patients into two groups based on whether their age was greater than or equal to 60 years and compared the PD-L1 expression status of CTCs between the groups. The results showed almost no difference between the PD-L1+ CTCs group and the PD-L1− CTCs group. Furthermore, the results also showed no significant difference in gender between these two groups of patients. After confirming that there were no significant differences in baseline data such as age and gender in relation to PD-L1 expression status in CTCs, we ruled out the possibility of significant bias in the study population. This allowed us to proceed with further analysis of pathological characteristics. So, we selected several clinicopathological characteristic indicators for correlation analysis.

Compared with PD-L1− CTCs patients, PD-L1+ CTCs patients presented with high-grade bladder cancer more frequently (*p* = 0.003). PD-L1+ CTCs patients did not show a higher incidence of muscle invasion contrary to PD-L1− CTCs patients (*p* = 0.144). However, patients with PD-L1+ CTCs also exhibited no significant differences from those with PD-L1− CTCs in terms of the presence or absence of lymph node metastasis (*p* = 0.189). When it comes to the specific subtype, there were almost no differences between patients with PD-L1+ CTCs and those with PD-L1− CTCs (*p* = 0.723), and, in terms of the LVI, there were still no significant differences between those two groups (*p* = 0.489). Furthermore, no significant differences were observed between patients with PD-L1+ CTCs and those with PD-L1− CTCs regarding either tumor size (*p* = 0.266) or the number of tumor lesions (*p* = 0.165). In terms of surgical modality, there was no statistically significant difference in the distribution of PD-L1+/− CTCs between the TURBT and RC groups (*p* = 0.697). Regarding pathological staging, there was no statistically significant difference in the distribution of PD-L1+/− CTCs between the low-stage and high-stage groups (*p* = 0.174) (Table 1).

### 3.3. Survival Prognosis

A total of 89 patients were included in the prognostic analysis. Among them, there were 74 (83.1%) male and 15 (16.9%) female, with a median age of 67 years. Patients with PD-L1+ CTCs numbered 17 (19.1%) and PD-L1− CTCs numbered 72 (80.9%). In addition, there were 24 (27.0%) patients with low-grade pathological grade and 65 (73.0%) with high-grade pathological grade. NMIBC patients numbered 63 (70.8%) and MIBC numbered 26 (29.2%). In total, five (5.6%) patients had lymph node metastasis. Moreover, there were nine (10.1%) patients with specific subtypes of bladder cancer, and seven (7.9%) patients showed LVI through the pathological findings of the bladder cancer. High pathological stage was present in 27 (30.3%) patients. The median follow-up duration was 48 months (IQR: 45.8–49.5).

**Figure 3:**
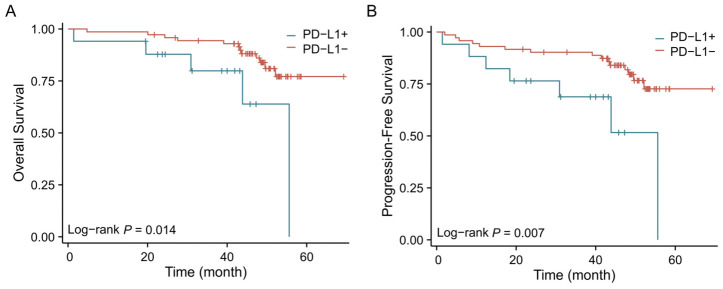
Prognostic analysis of PD-L1+/− in the study population. (**A**) Overall survival (OS), Kaplan–Meier curve of PD-L1+/− in the cohort. (**B**) Progression-free survival (PFS), Kaplan–Meier curve of PD-L1+/− in the cohort.

First, we used Kaplan–Meier methods to analyze whether the PD-L1 expression status in CTCs have a significant effect on OS or PFS in bladder cancer. The Kaplan–Meier curve demonstrated significantly shorter OS in PD-L1+ CTCs patients compared to PD-L1− CTC patients (*p* = 0.014) (Figure 3A). Regarding PFS, Kaplan–Meier curves showed a similar trend to the PFS, specifically demonstrating that patients with PD-L1+ CTCs showed a significantly lower survival rate than those with PD-L1− CTCs (*p* = 0.007) (Figure 3B).

Next, we investigated the primary determinant of poor prognosis in patients with bladder cancer. Results indicated that PD-L1 in CTCs (HR = 3.602 (1.217–10.656), *p* = 0.021) and Lymph vascular Invasion (HR = 4.072 (1.143–14.502), *p*= 0.03) were identified as risk factors for OS in bladder cancer patients. Further, Firth penalized Cox regression analysis revealed that PD-L1 in CTCs (HR = 4.696 (1.477–13.596), *p* = 0.011) and Lymph vascular Invasion (HR = 6.166 (1.522–19.776), *p* = 0.014) were independent risk factors for OS in bladder cancer (Table 2).

When it comes to PFS, the Cox regression analysis results showed that Age (HR = 7.853 (1.055–58.435), *p* = 0.044), Neoadjuvant therapy (HR = 3.483 (1.247–9.733), *p* = 0.017), Maintenance intravesical instillation (HR = 0.324 (0.116–0.902), *p* = 0.031), PD-L1 in CTCs (HR = 3.385 (1.332–8.604), *p* = 0.01), and Lymph node metastasis (HR = 4.053 (1.187–13.834), *p* = 0.025) were identified as risk factors for PFS. Further, Firth penalized Cox regression analysis revealed that Age (HR = 7.481 (1.760–74.382), *p* = 0.003) and Lymph node metastasis (HR = 5.584 (1.143–26.558), *p* = 0.035) were independent risk factors for PFS in bladder cancer (Table 3).

### 3.4. Subgroup Analysis

Finally, we performed subgroup classification based on distinct pathological features and tried to evaluate whether there was an association between PD-L1 expression in CTCs and PFS or OS within these subgroups. Given that the small sample sizes in some subgroups could lead to significant result bias, we only selected a part of bladder cancer subgroup patients for the relevant PFS and OS analysis.

Among them, there were 8 (30.8%) PD-L1+ CTCs patients and 18 (69.2%) PD-L1− CTC patients in the muscle-invasive bladder cancer (MIBC) subgroup. The number of OS events was two and three, respectively; the number of PFS events was three and three, respectively. Meanwhile, in the non-muscle-invasive bladder cancer (NMIBC) subgroup, 54 (85.7%) patients had PD-L1− CTCs and nine (14.3%) patients had PD-L1+ CTCs. The number of OS events was nine and three, respectively; the number of PFS events was 12 and four, respectively. When it comes to high-grade bladder cancer subgroups, there were 48 (73.8%) patients with PD-L1− CTCs and 17 (26.2%) patients with PD-L1+ CTCs. The number of OS events was seven and five, respectively; the number of PFS events was nine and seven, respectively.

**Figure 4:**
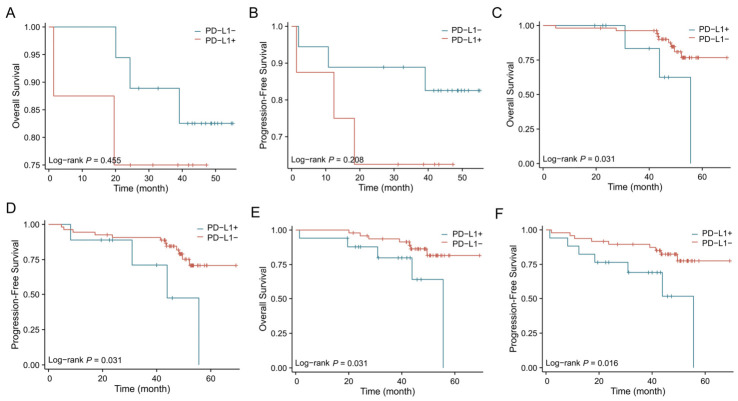
Kaplan–Meier estimates of survival stratified by PD-L1+/− CTCs in subgroups. (**A**) OS in MIBC subgroup. (**B**) PFS in MIBC subgroup. (**C**) OS in NMIBC subgroup. (**D**) PFS in NMIBC subgroup. (**E**) OS in high-grade bladder cancer. (**F**) PFS in high-grade bladder cancer. MIBC—muscle-invasive bladder cancer; NMIBC—non–muscle-invasive bladder cancer; PFS—progression-free survival; OS—overall survival; HR—hazard ratio.

The Kaplan–Meier curves demonstrated no difference in OS (HR = 1.948 (0.261–14.550), *p* = 0.455) (Figure 4A) or PFS (HR = 2.674 (0.443–16.144), *p* = 0.208) (Figure 4B) between PD-L1+ CTC patients and PD-L1− CTC patients in the MIBC cohort. In NMIBC patients, PD-L1+ CTCs showed a striking trend towards reduced OS compared with PD-L1− CTCs (HR= 0.266 (0.034–2.105), *p* = 0.031) (Figure 4C). And NMIBC patients with PD-L1+ CTCs had poor PFS compared with PD-L1− CTC patients (HR = 0.312 (0.058–1.670), *p* = 0.031) (Figure 4D). The results indicated significant differences in OS (HR = 0.307 (0.070–1.339), *p* = 0.031) and PFS (HR = 0.322 (0.095–1.096), *p* = 0.016) between PD-L1+ CTC and PD-L1− CTC groups in the subgroup of patients with high-grade urothelial bladder cancer (Figure 4E,F).

## 4. Discussion

In this study, we focused on the value of PD-L1 expression in CTCs in relation to the clinical and pathological characteristics of bladder cancer, as well as its roles in guiding prognosis and predicting treatment outcomes. We found that PD-L1+ CTCs were significantly associated with bladder cancer grade, which was specifically manifested by the fact that patients with PD-L1+ CTCs had a higher pathological grade of bladder cancer compared with those with PD-L1− CTCs. Moreover, the presence of PD-L1+ CTCs was associated with poor prognosis in patients, which was specifically manifested by the fact that bladder cancer patients with detectable PD-L1+ CTCs had significantly shorter OS than those with PD-L1− CTCs. Meanwhile, to some extent, subgroup analysis revealed that PD-L1+ CTCs were an indicator of poorer PFS and OS compared with PD-L1− CTCs, with a significant difference between the two groups in the non-muscle-invasive bladder cancer (NMIBC) population and patients with high-grade urothelial bladder cancer.

Metastasis is an early event in patients with aggressive cancers, often occurring before the primary tumor is clinically detected. Studies have shown that even patients with small, early-stage tumors may experience early metastasis to distant sites. Metastasis typically results from the spread of cancer cells through the bloodstream to distant non-malignant tissues. Studies have indicated that CTCs are closely associated with the hypoxic tumor microenvironment, and the presence of cancer stem cell-like properties in CTCs also suggests an unfavorable prognosis for patients [23]. The penetration of tumor cells through the basement membrane into blood vessels to form CTC is considered a key factor in metastasis [24]. Consequently, the presence of CTC indicates the potential for metastasis. Studies have shown that CTCs achieve immune escape through mechanisms including antigen loss, secretion of immunosuppressive factors, expression of immunological checkpoint (IC) proteins, and production of extracellular vesicles, ultimately leading to clinical outcomes such as tumor progression and treatment resistance [25]. Jin D et al. reported that CTC positivity correlates with tumor multiplicity, tumor size, and grade/stage classification in bladder cancer [14]. Our previous study similarly found that the number of CTCs is closely associated with response to neoadjuvant chemotherapy. Specifically, CTCs exhibit high predictive value for the sensitivity of neoadjuvant chemotherapy in bladder cancer, which can provide a certain reference for determining whether RC is indicated for patients [17]. Previous research conducted by Mustafa Abdo et al. in patients with non-small cell lung cancer (NSCLC) reported a moderate overall consistency of PD-L1 positivity between histological tumor tissues and CTCs [26]. This finding indicates that the detection of PD-L1 expression on CTCs could, to a certain extent, improve the predictive efficiency of tumor tissue PD-L1 status. Although CTC PD-L1 detection has not yet been integrated into routine clinical practice, we believe that further investigations clarifying its correlation with PD-L1 expression in primary tumor lesions will help validate CTC PD-L1 as a novel and promising biomarker for evaluating pathological characteristics and predicting clinical prognosis in cancer patients.

The clinical application of immune checkpoint inhibitors (ICI) has drawn researchers’ attention to the expression of IC proteins on CTCs. Studies have revealed that CTCs possess the capability to express immune checkpoint proteins, which interact with immune cell receptors and suppress immune cell activity [25]. In cancers such as breast cancer and prostate cancer, CTCs express PD-L1 and CTLA-4, thereby promoting tumor growth [20,27]. We employed SE-iFISH technology and a series of cell-specific markers to isolate CTCs identified as CD45−, CD31−, DAPI+, and CEP8+. Subsequently, PD-L1 expression on the surface of these CTCs was evaluated using a PD-L1 specific antibody-conjugated fluorescent antibody. We discovered that CTCs in the blood of bladder cancer patients also express PD-L1, and the presence of PD-L1+ CTCs is associated with higher tumor grade in bladder cancer patients. High expression of PD-L1 can bind to PD-1 on the surface of T cells, inhibiting T cell activation and cytotoxicity. This allows tumor cells expressing PD-L1 to gain a survival advantage, gradually becoming the dominant clone under immune selective pressure [28].

In this study, approximately 50% of patients with PD-L1+ CTCs were pathologically confirmed to have muscle-invasive disease. And PD-L1+ CTCs were found to be significantly associated with poor PFS. Suzanne L. et al. considered that PD-L1 may inhibit T cell function by binding to the PD-1 receptor on the T cell surface, thereby weakening anti-tumor immunity, fostering an immunosuppressive microenvironment, and ultimately leading to poor prognosis [29]. Not an isolated case, Morelli, M. B., et al. also reported that high PD-L1 mRNA reduces the RFS in NMIBC patients [29]. As indicated above, the expression of PD-L1 on CTC is associated with unfavorable pathological features. Kong D et al. conducted a meta-analysis, revealing that PD-L1+ CTCs are associated with poor prognosis in various tumor types, including NSCLC, HNSCC, prostate cancer, and melanoma, and the pooled HR for OS confirmed PD-L1+ CTCs as an indicator of poor prognosis in patients not receiving ICI therapy [30]. Strati et al. reported that in patients with head and neck squamous cell carcinoma, PD-L1 overexpression in CTC was associated with significantly shorter PFS (*p* = 0.001) and OS (*p* < 0.001) [30]. Xiaoling Wang et al. proposed that liquid biopsy markers such as CTCs, circulating tumor DNA (ctDNA), and extracellular vehicles (EVs) each have their own advantages. For instance, CTCs can reflect the overall characteristics of a tumor, ctDNA is widely used to assist in disease diagnosis, treatment, and prognosis, while EVs contain a variety of tumor-derived substances. Despite their different focuses, they are strongly complementary to a certain extent [31]. This suggests that the detection of PD-L1 on CTC surfaces can be linked with other existing liquid biopsy methods. We look forward to more researchers exploring the specific connections between the PD-L1 indicator on CTC surfaces and other liquid biopsy methods, or even more detection modalities, in the future. Bergmann et al. utilized the CellSearch^®^ system to evaluate 49 patients with advanced urothelial carcinoma (UC) and found that PD-L1 positive CTCs were associated with shorter overall survival (OS). However, the aforementioned studies were limited to advanced or metastatic disease, had small sample sizes, and did not directly compare their findings with established prognostic models. Our study is not limited to patients with advanced bladder cancer (BCa), and the sample size is relatively larger. Although there is still a potential risk of statistical bias and result deviation due to the low incidence of positive events, this still provides a certain reference for the theory related to the detection of PD-L1 on CTC surfaces in BCa patients.

The current study has several limitations. First, the relatively small number of endpoint events may lower the statistical power of survival analysis and increase random errors in the results. We will continue patient follow-up to collect sufficient data for further analyses of prognostic associations. Second, Firth penalized Cox regression analysis was adopted to enhance the robustness of the survival model. Cross-validation using multiple alternative statistical methods could further optimize model fitting and improve the reliability of our findings. Third, incomplete collection of baseline clinical data prevented adjustment for several confounding factors, which may partially interfere with the quantitative assessment of associations between exposure variables and clinical outcomes. Fourth, the simplified criteria for defining PD-L1+CTCs may reduce the accuracy of effect estimation for this biomarker. Lastly, only one liquid biopsy platform was utilized in the present work. In future investigations, we aim to enlarge the sample size and conduct prospective studies with multiple detection assays and complementary statistical validations to verify and refine the conclusions of this research.

## 5. Conclusions

Based on SE-iFISH strategy, the present study researches for the relationship between PD-L1 expression status on CTCs and pathological stage, grade, and clinical outcome in bladder cancer. To some extent, our findings suggested that the PD-L1 expression status on CTCs may have diverse potential to guide the diagnosis, prognosis prediction, and therapeutic decisions in bladder cancer. Specifically, it indicated that the presence of PD-L1+ CTCs in patients with bladder cancer may be associated with high grade disease and poor OS. But further analytical validations are still required.

## Figures and Tables

**Table 1:** Correlation analysis of PD-L1 expression with various factors in CTC+ patients.

Characteristics *n* (%)	PD-L1− CTCs (*n* = 90)	PD-L1+ CTCs (*n* = 19)	*p* Value
Age			
<60 years	23 (82.1)	67 (82.7)	>0.9
≥60 years	5 (17.9)	14 (17.3)	
Gender			
Male	77 (82.8)	13 (81.3)	>0.9
Female	16 (17.2)	3 (18.8)	
Muscularis propria invasion,			
Absence	63 (86.3)	10 (13.7)	0.144
Presence	27 (75)	9 (25)	
Lymph Node Metastasis			
Absence	84 (84)	16 (16)	0.189
Presence	6 (66.7)	3 (33.3)	
Histological Grade			
Low-grade	28 (100)	0 (0)	0.003
High-grade	62 (76.5)	19 (23.5)	
Special Histological Subtype			
Absence	78 (83)	16 (17)	0.723
Presence	12 (80)	3 (20)	
Lymphovascular Invasion (LVI)			
Absence	81 (81.8)	18 (18.2)	0.489
Presence	9 (90)	1 (10)	
Tumor Size			
Maximum diameter < 3 cm	68 (85)	12 (15)	0.266
Maximum diameter ≥ 3 cm	22 (75.9)	7 (24.1)	
Tumor Multifocality			
Single	58 (86.6)	9 (13.4)	0.165
Multiple	32 (76.2)	10 (23.8)	
Surgical modality			
TURBT	61 (83.6)	12 (16.4)	0.697
RC	29 (80.6)	7 (19.4)	
Pathological staging			
Low stage	62 (86.1)	10 (13.9)	0.174
High stage	28 (75.7)	9 (24.3)	

**Table 2:** Univariate and multivariate Cox regression analyses for overall survival (OS).

Variable	OS (*n* = 89)			
	Univariate Analysis		Multivariate Analysis	
	HR (95%CI)	*p* Value	HR (95%CI)	*p* Value
Age (≥60 y versus <60 y)	5.531 (0.733–41.753)	0.097		
Gender (Male versus Female)	0.511 (0.117–2.242)	0.374		
Tumor size (<3 cm versus ≥3 cm)	1.446 (0.465–4.503)	0.524		
Tumor number (Solitary versus Multiple)	1.896 (0.825–4.355)	0.132		
Neoadjuvant therapy (No versus Yes)	2.632 (0.728–9.511)	0.14		
Maintenance intravesical instillation (Yes versus No)	0.488 (0.17–1.406)	0.184		
Adjuvant systemic treatment (No versus Yes)	1.022 (0.291–3.591)	0.973		
PD-L1 in CTCs (PD-L1+ versus PD-L1−)	3.602 (1.217–10.656)	0.021	4.696 (1.477–13.596)	0.011
Muscularis propria invasion (Positive versus Negative)	1.545 (0.531–4.494)	0.425		
Lymph node metastasis (Positive versus Negative)	3.326 (0.751–14.733)	0.113		
Histological Grade (High versus Low)	1.164 (0.408–3.322)	0.776		
Special Subtype (Positive versus Negative)	2.565 (0.728–9.039)	0.143		
Lymph vascular Invasion (Positive versus Negative)	4.072 (1.143–14.502)	0.03	6.166 (1.522–19.776)	0.014
surgical modality	2.357 (0.874–6.357)	0.09		
pathological staging	1.392 (0.48–4.034)	0.542		

**Table 3:** Univariate and multivariate Cox regression analyses for progression-free survival (PFS).

Variable	PFS (*n* = 89)			
	Univariate Analysis		Multivariate Analysis	
	HR (95%CI)	*p* Value	HR (95%CI)	*p* Value
Age (≥60 y versus <60 y)	7.853 (1.055–58.435)	0.044	7.481 (1.760–74.382)	0.003
Gender (Male versus Female)	0.631 (0.186–2.142)	0.46		
Tumor size (<3 cm versus ≥3 cm)	0.934 (0.314–2.781)	>0.9		
Tumor number (Solitary versus Multiple)	1.161 (0.53–2.541)	0.709		
Neoadjuvant therapy (No versus Yes)	3.483 (1.247–9.733)	0.017	1.718 (0.440–5.261)	0.401
Maintenance intravesical instillation (Yes versus No)	0.324 (0.116–0.902)	0.031	0.444 (0.147–1.174)	0.103
Adjuvant systemic treatment (No versus Yes)	1.368 (0.501–3.739)	0.541		
PD-L1 in CTCs (PD-L1+ versus PD-L1−)	3.385 (1.332–8.604)	0.01	2.074 (0.681–5.500)	0.186
Muscularis propria invasion (Positive versus Negative)	1.213 (0.468–3.146)	0.691		
Lymph node metastasis (Positive versus Negative)	4.053 (1.187–13.834)	0.025	5.584 (1.143–26.558)	0.035
Histological Grade (High versus Low)	1.226 (0.477–3.149)	0.672		
Special Subtype (Positive versus Negative)	2.344 (0.787–6.986)	0.126		
Lymph vascular Invasion (Positive versus Negative)	2.643 (0.773–9.039)	0.121		
surgical modality	2.113 (0.888–5.03)	0.091		
pathological staging	1.105 (0.427–2.859)	0.836		

## Data Availability

The data presented in this study are available on request from the corresponding author due to patient privacy and ethical restrictions.

## Supplementary Materials

The following supporting information can be downloaded at: https://www.mdpi.com/article/10.3390/diagnostics16121776/s1, Table S1: Correlation Analysis of CTC with Various Factors in Patients with bladder cancer.
