# Supplementary material for: PD-L1+ CTCs Are Associated with Adverse Pathological Features and Unfavorable Prognosis in Bladder Cancer
Source: Diagnostics (Basel). 2026 Jun 9;16(12):1776. doi: 10.3390/diagnostics16121776 (PMC13298106; doi:10.3390/diagnostics16121776)
Supplement: Supplementary file 1 [file diagnostics-16-01776-s001.zip › diagnostics-4205356-supplementary.pdf]

Table S1. Correlation Analysis of CTC with Various Factors in Patients with bladder cancer.

characteristics, n (%)	non-CTCs(n=12)	CTCs(n=109)	*P* value
Age			
<60 years	6(17.6)	6(6.9)	0.15
≥60 years	28(82.4)	81(93.1)	
Gender			
Male	10(9.7)	2(11.1)	> 0.9
Female	93(90.3)	16(88.9)	
Muscularis propria invasion,			
Absence	8(66.7)	4(33.3)	> 0.9
Presence	73(67)	36(33)	
Lymph Node Metastasis			
Absence	11(91.7)	1(8.3)	> 0.9
Presence	100(91.7)	9(8.3)	
Histological Grade			
Low-grade	2(16.7)	10(83.3)	0.475
High-grade	28(25.7)	81(74.3)	
Special Histological Subtype			
Absence	10(83.3)	2(16.7)	0.676
Presence	94(86.2)	15(13.8)	
Lymphovascular Invasion (LVI)			
Absence	11(91.7)	1(8.3)	> 0.9
Presence	99(90.8)	10(9.2)	
Tumor Size			
Maximum diameter<3cm	9(75)	3(25)	> 0.9
Maximum diameter≥3cm	80(73.4)	29(26.6)	
Tumor Multifocality			
single	7(58.3)	5(41.7)	0.886
multiple	67(61.5)	41(37.6)	
Surgical modality			
TURBT	8(9.9)	73(90.1)	> 0.9
RC	4(10)	36(90)	
Pathological staging			
Low stage	8(10)	72(90)	> 0.9
High stage	4(9.8)	37(90.2)	
